# Adiponectin promotes muscle regeneration through binding to T-cadherin

**DOI:** 10.1038/s41598-018-37115-3

**Published:** 2019-01-09

**Authors:** Yoshimitsu Tanaka, Shunbun Kita, Hitoshi Nishizawa, Shiro Fukuda, Yuya Fujishima, Yoshinari Obata, Hirofumi Nagao, Shigeki Masuda, Yuto Nakamura, Yuri Shimizu, Ryohei Mineo, Tomoaki Natsukawa, Tohru Funahashi, Barbara Ranscht, So-ichiro Fukada, Norikazu Maeda, Iichiro Shimomura

**Affiliations:** 1 Department of Metabolic Medicine, Graduate School of Medicine, Osaka University, Osaka, Japan; 2 Department of Adipose Management, Graduate School of Medicine, Osaka University, Osaka, Japan; 3 Department of emergency & intensive care, Yodogawa Christian Hospital, Osaka, Japan; 4 Department of Metabolism and Atherosclerosis, Graduate School of Medicine, Osaka University, Osaka, Japan; 5 Sanford Burnham Prebys Medical Discovery Institute, NIH-designated Cancer Center, Development, Aging and Regeneration Program, La Jolla, CA USA; 6 Laboratory of Molecular and Cellular Physiology, Graduate School of Pharmaceutical Sciences, Osaka University, Osaka, Japan

## Abstract

Skeletal muscle has remarkable regenerative potential and its decline with aging is suggested to be one of the important causes of loss of muscle mass and quality of life in elderly adults. Metabolic abnormalities such as obesity were linked with decline of muscle regeneration. On the other hand, plasma levels of adiponectin are decreased in such metabolic conditions. However, plasma levels of adiponectin have been shown to inversely correlate with muscle mass and strength in elderly people especially with chronic heart failure (CHF). Here we have addressed whether adiponectin has some impact on muscle regeneration after cardiotoxin-induced muscle injury in mice. Muscle regeneration was delayed by angiotensin II infusion, mimicking aging and CHF as reported. Adiponectin overexpression *in vivo* decreased necrotic region and increased regenerating myofibers. Such enhanced regeneration by excess adiponectin was also observed in adiponectin null mice, but not in T-cadherin null mice. Mechanistically, adiponectin accumulated on plasma membrane of myofibers both in mice and human, and intracellularly colocalized with endosomes positive for a multivesicular bodies/exosomes marker CD63 in regenerating myofibers. Purified high-molecular multimeric adiponectin similarly accumulated intracellularly and colocalized with CD63-positive endosomes and enhanced exosome secretion in differentiating C2C12 myotubes but not in undifferentiated myoblasts. Knockdown of T-cadherin in differentiating C2C12 myotubes attenuated both adiponectin-accumulation and adiponectin-mediated exosome production. Collectively, our studies have firstly demonstrated that adiponectin stimulates muscle regeneration through T-cadherin, where intracellular accumulation and exosome-mediated process of adiponectin may have some roles.

## Introduction

Skeletal muscle is a post-mitotic tissue with a very low turnover rate^1^, however, has a strong regenerating ability after injury. The age-related reduction in muscle regenerating ability is suggested to contribute to the loss of muscle mass in elderly people^2^, although there are some conflicting reports showing defect of regeneration affected muscle fibrosis but not muscle mass in mice^3,4^. Preserving muscle regeneration ability may slow the development of this syndrome.

Regeneration of skeletal muscle depends on muscle stem cells, named satellite cells, which are activated upon muscle damage to expand and to differentiate into myogenic cells that regenerate damaged muscle^5,6^. Such regenerative responses are gradually lost in aged muscle, probably because of the reduced number and attenuated function of satellite cells^2,7,8^. Importantly, the regenerating capacity is blunted also by metabolic abnormalities such as obesity^9^, type 2 diabetes^10^ and hypertension^11,12^.

Adiponectin (APN) is a secreting factor from adipocytes, and its circulating level decreases in metabolic abnormalities such as obesity, diabetes and hypertension. APNs are assembled intracellularly and secreted as trimer, hexamer and high molecular multimer^13,14^. High molecular multimer APN is thought as the active form to exert various pleiotropic effects from number of clinical studies^15–18^. APN accumulates in tissues through interaction with T-cadherin (T-cad), a unique glycosylphosphatidylinositol (GPI)-anchored cadherin^19–21^, whose single nucleotide polymorphism correlates strongly with plasma APN level and cardiovascular diseases^22–27^. We and others reported that cardiovascular protections by APN required T-cad in several mouse models^19,20,28^. Furthermore, we recently reported that T-cad specifically recognized high molecular multimer APN with high affinity^29^ and uniquely mediated accumulation in endosomes and ceramide-reducing effect of APN by stimulating exosome-biogenesis and secretion^30^.

Recent clinical studies have shown that plasma APN levels inversely correlated with muscle weakness in elderly people^31,32^, muscle mass and strength especially in heart failure patients^33^. T-cad is expressed abundantly in skeletal muscle in addition to above mentioned vascular endothelium and heart^21^. APN functions through T-cad on muscle homeostasis especially on its regenerating potential warrants investigation in detail.

Here, we have tested the effect of APN on muscle regeneration in mice. First, we found that APN accumulated on plasma membrane of myofibers, and in CD63-positive endosomes of regenerating myotubes. Functionally, APN overexpression *in vivo* decreased necrotic region and increased regenerating myofibers in impaired muscle regeneration model. Such enhanced regeneration by excess APN was also observed in APN null mice, but not in T-cad null mice. Our studies have firstly demonstrated that APN enhances muscle regeneration through T-cad.

## Results

### APN accumulation in regenerating muscle fibers

**Figure 1:**
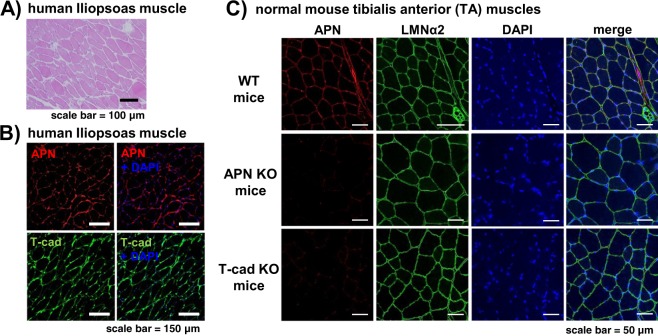
Localization of APN and T-cad in intact human and mouse muscle. A series of paraffin sections of human iliopsoas muscle autopsy specimen was stained with H&E (**A**), with anti-human APN and with anti-human T-cad (**B**). Cell nuclei were counterstained with DAPI. Scale bars, 100 μm for (**A**), 150 μm for (**B**). (**C**) Confocal immunofluorescence micrographs of tibialis anterior (TA) muscle of WT, adiponectin KO (APN KO), and T-cadherin KO (T-cad KO) mice. Muscle tissues were stained with anti-adiponectin (APN; red), anti-laminin α2 (LMNα2; green). Cell nuclei were counterstained with DAPI (blue). Scale bars, 50 μm.

Firstly, we have characterized a human iliopsoas muscle autopsy specimen (Fig. 1). H&E staining results indicated almost intact muscle with tightly associated myofibers (Fig. 1A). Immunohistochemical studies showed that APN was found at the surface of myofibers and well colocalized with T-cad in these serial sections (Fig. 1B). Such accumulation of APN at the surface of myofibers was also observed in WT mouse tibialis anterior (TA) muscle, in which APN well colocalized with basal membrane marker, laminin α2 (Fig. 1C upper panels). However, the accumulation of APN was not observed in T-cad KO mouse (Fig. 1C bottom panels), similarly in APN KO mouse (Fig. 1C middle panels), suggesting that such ectopic accumulation of APN other than its producing adipose tissues requires T-cad.

**Figure 2:**
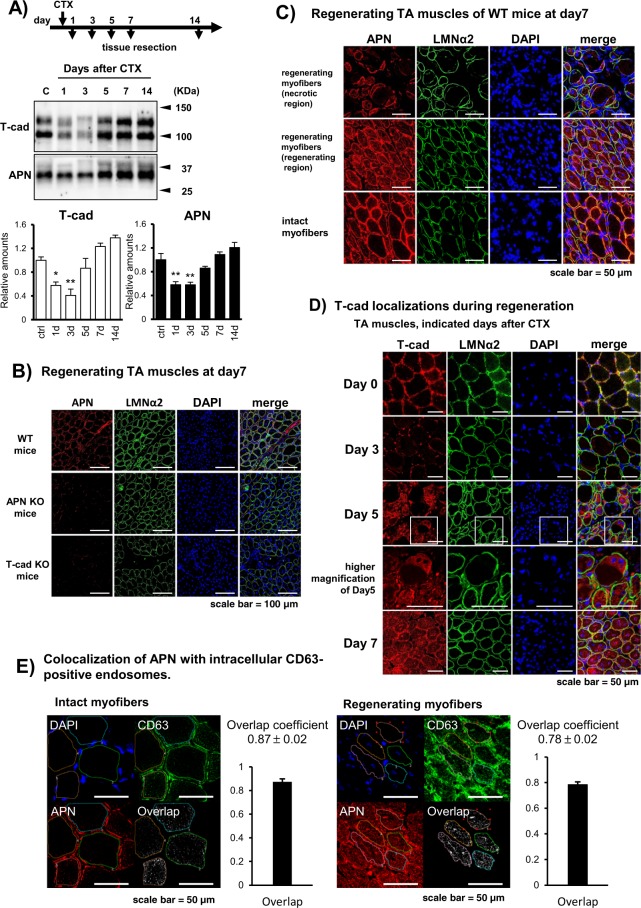
Accumulation of APN in tibialis anterior muscle during regeneration. (**A**) Western blots of tibialis anterior (TA) muscle tissue lysates obtained at indicated periods after CTX injection. Equal amounts of protein were separated by SDS-page. T-cad (105 and 130 kDa) and APN (30 kDa) were quantified and shown in the right panel. *n* = *3*. Data are mean ± s.e.m., *P < 0.05; **P < 0.01; Dunnett’s test. (**B**–**D**) Confocal immunofluorescence micrographs of TA muscle stained with anti-laminin α2 (LMNα2; green). Cell nuclei were counterstained with DAPI (blue). (**B**) TA muscle of WT, adiponectin KO (APN KO), and T-cadherin KO (T-cad KO) mice at day7 after CTX-injury. Anti-adiponectin (APN; red). Scale bars, 100 μm. (**C**) Characteristic regions in TA muscle of WT mice. Muscle tissues at 7 days after CTX injection. Upper panels; emerging crescentic myofibers with centered nucleus in necrotic region with balloon like myofibers lacking nucleus. Middle panels; regenerating myofibers in actively regenerating region with dense smaller myofibers with centered nucleus. Bottom panels; intact myofibers with eccentric nucleus. Anti-adiponectin (APN; red). Scale bars, 50 μm. (**D**) TA muscle tissues of WT mice obtained at indicated days after CTX injection. Higher magnifications of day5 were included. Anti-T-cadherin (T-cad; red). Scale bars, 50 μm. (**E**) Overlap coefficient of APN and CD63 in intact myofibers (upper panels) and regenerating myofibers (lower panels) at 7 days after CTX injection was calculated as described in *Methods*. Data are mean ± s.d., n = 4 (intact myofibers), n = 5 (regenerating myofibers).

Next, we compared the protein amount of APN and T-cad in TA muscles during the process of skeletal muscle regeneration following cardiotoxin (CTX)-injury (Fig. 2). T-cad protein expression once decreased between day1 and day3 after CTX injection, and then it turned to increase to normal level after day7 (Fig. 2A). The amount of APN protein in TA muscles also decreased after CTX injection and recovered to normal levels, in line with the change of T-cad (Fig. 2A).

To examine the localization of APN in the regenerating muscle, TA muscles from WT were resected 7days after CTX injection and then subjected to immunofluorescence. APN accumulation significantly decreased in such regenerating muscle at day7 after CTX-injury in T-cad KO mice than in WT mice, indicating T-cad dependent mechanism for APN accumulation in myofibers (Fig. 2B). CTX injury is known to induce massive necrosis of myofibers. Upon necrosis, migrating neutrophils, and macrophages elicit inflammation^34^. This activates muscle stem cells, known as satellite cells residing under the basal lamina, to grow into regenerating myofibers^35^. Such actively regenerating myofibers can be distinguished from intact or already regenerated mature fibers by their characteristic centrally located nuclei. We precisely investigated different phases of regenerating myofibers if APN might accumulate differentially (Figs 2C and S1 for higher magnifications). APN was found intracellularly in some endosome structures as well as at the cell surface in emerging crescentic myotubes in necrotic region with balloon like myofibers lacking nucleus (Fig. 2C upper panels), and in actively regenerating myotubes with centrally located nuclei (Fig. 2C middle panels), while APN was not found intracellularly but only at the surface of myofibers in intact fibers with peripherally located nuclei (Fig. 2C bottom panels). In a time-course experiment, T-cad at the surface of myofibers in intact muscle was almost lost at day3 after CTX-injury. Regenerating myofibers which appeared at day5 and grew at day7 had intracellularly distributed T-cad in some endosome structures as well as in cell surface (Fig. 2D). Immunofluorescence colocalization experiments indicated that APN highly colocalized with a multivesicular body/exosome marker CD63 (Fig. 2E), suggesting endocytosis of APN into CD63-positive endosomes.

### Effect of APN on muscle regeneration

**Figure 3:**
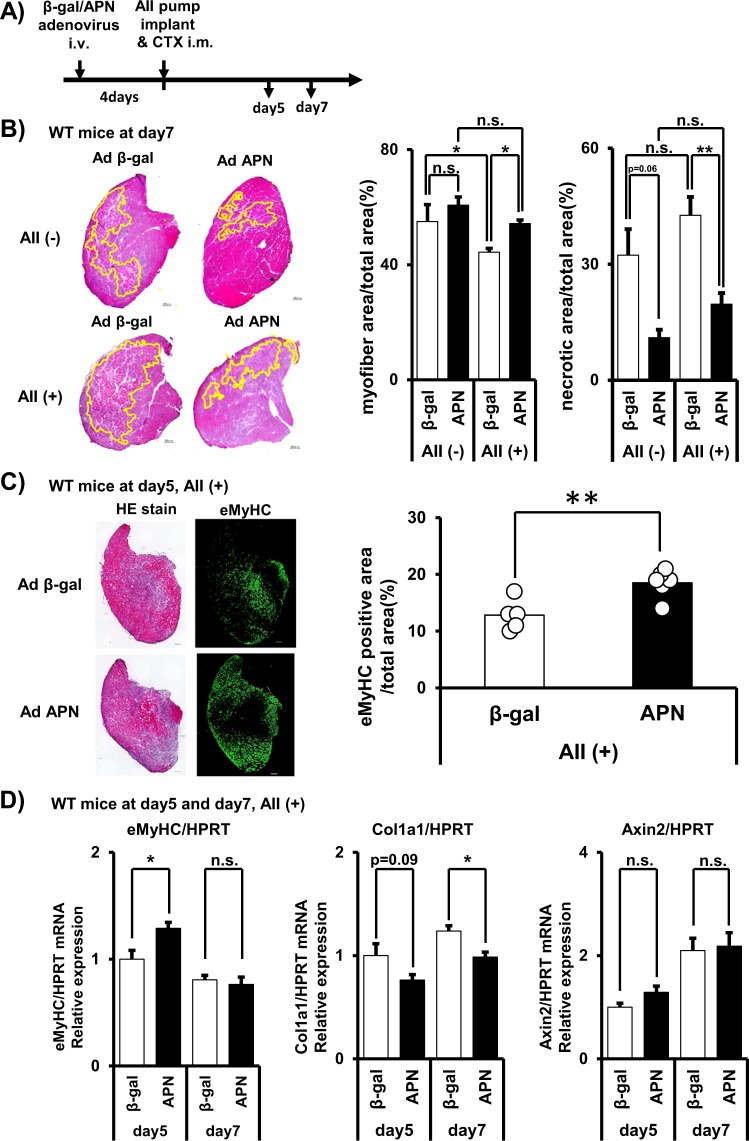
Effect of adiponectin overexpression on muscle regeneration. (**A**) Overview of the experiment for assessing the effects of adiponectin overexpression on muscle regeneration. (**B**) Representative H&E staining of tibialis anterior (TA) muscle at 7 days after CTX injection. Scale bars, 200 μm. Myofiber area (%) (myofiber area/total area) was quantified from H&E stains. Residual necrotic region area (%) (necrotic area/total area) was identified and quantified from H&E staining by a method described in *Methods*. Data are mean ± s.e.m., n = 3 (AII−), n = 6 (AII+), *P < 0.05; **P < 0.01, Tukey’s test. Raw image and identified necrotic region of all sections were in Fig. S3. (**C**) Immunofluorescent staining of embryonic myosin heavy chain (eMyHC) in WT mice. eMyHC positive myofiber area was quantified and shown in right. (**D**) mRNA expression of eMyHC, collagen 1a1 (Col1a), and AXIS inhibition protein 2 (Axin2). Data are mean ± s.e.m., n = 5 (day5, APN), n = 6 (others), n.s; not significant; *P < 0.05.

Next, we have tested the effect of APN on muscle regeneration in mice. We administered APN-expressing adenovirus intravenously. Four-days after infection, CTX was injected into mouse TA muscles to induce muscle regeneration process (Fig. 3A). APN overexpression successfully increased serum APN concentrations (Fig. S2A). Although necrotic region area (%) in total muscle section area showed tendency of reduction by APN-overexpression after 7 days of CTX-injury (p = 0.06), myofiber area (%) in total muscle section area was not changed compared to control β-gal-overexpression group (Fig. 3B). Since C57BL/6J mouse strain is known to have a very high regenerating capacity, we performed the experiment under angiotensin II (AII)-infusion using implanted osmotic pump, which can partially mimic aging^36^ and chronic heart failure condition (Fig. 3A)^11,12^. AII-infused mice showed significant reduction of the ratio of myofiber area (%) in response to CTX-injury (Fig. 3B), showing that AII impaired regenerating capacity. Under such suppressed regenerating condition, APN overexpression significantly increased the myofiber area (%) and decreased remaining necrotic region area (%), compared to those of β-gal-overexpression (Fig. 3B). APN overexpression also increased the eMyHC positive area (%) at 5 days after CTX-injury (Fig. 3C) and eMyHC mRNA expression (Fig. 3D), and decreased collagen 1a1 (Col1a1) mRNA expression (Fig. 3D), further confirming the enhancing role of APN in muscle regeneration. On the other hand, one of the measures of AII signaling, Axin2 mRNA expression was stimulated by AII-infusion (Fig. S2B) and not significantly altered by APN overexpression (Fig. 3D), suggesting that APN did not attenuate AII signaling itself. Canonical AII-signaling including phosphorylated ERK, p38MAPK, p65NFκB, and Nox2 protein was not significantly altered by AII-infusion nor by APN (Fig. S2C-F).

### Effect of APN overexpression on muscle regeneration in genetic absence of APN or T-cad

**Figure 4:**
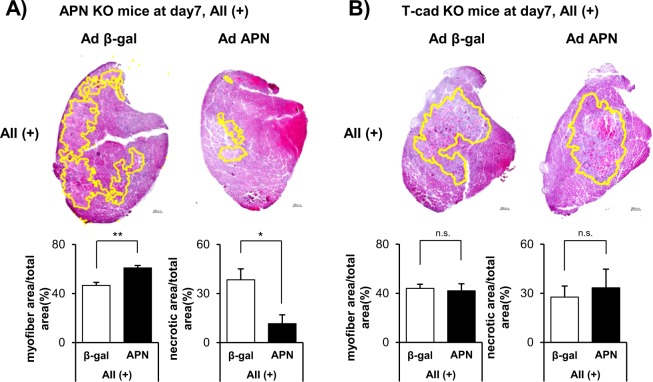
Effect of adiponectin overexpression on muscle regeneration in genetic absence of APN or T-cad. H&E staining of tibialis anterior (TA) muscle at 7 days after CTX injection in APN KO mice (**A**) or in T-cad KO mice (**B**). Representative photographs were shown in left. Scale bars, 200 μm. Myofiber area (%) (myofiber area/total area) is quantified from H&E stains. Residual necrotic region area (%) (necrotic area/total area) is quantified from H&E staining by a method described in *Methods*. Raw image and identified necrotic region of all sections were in Fig. S3. Data are mean ± s.e.m., n = 6 (APN), n = 6 (β-gal), n.s; not significant; *P < 0.05.

Next, we conducted the same AII-loaded CTX-induced muscle regeneration experiment in APN KO mice and T-cad KO mice (Fig. 4). Loss of APN or T-cad did not significantly affect muscle regeneration, when we compared myofiber area (%) and necrotic area (%) between β-gal overexpressing WT mice (Fig. 3B) and APN KO mice (Fig. 4A) or T-cad KO mice (Fig. 4B). APN overexpression significantly improved muscle regeneration in APN KO mice, as judged by enhanced myofiber area and decreased necrotic region area (%) (Fig. 4A). Importantly, such improvement was not observed in T-cad KO mice, demonstrating the importance of T-cad in APN-mediated effect on muscle regeneration (Fig. 4B).

### Differentiation dependent intracellular localization of APN in C2C12

**Figure 5:**
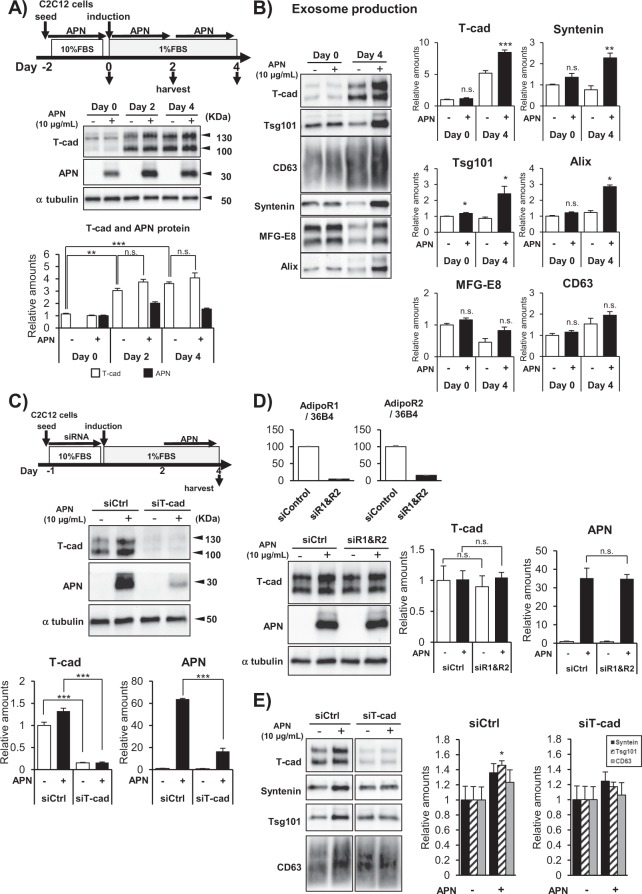
APN accumulation and exosome secretion. (**A**,**B**) Differentiation dependence of APN-accumulation and exosome secretion. (**A**) Proteins in cell lysates. Experimental scheme was summarized. Blots for T-cad (130 kDa and 100 kDa), APN (30 kDa) and α tubulin (48 kDa) were shown. Data are mean ± s.e.m., n = 3, n.s; not significant; **P < 0.01; ***P < 0.001; Tukey’s test. (**B**) Undifferentiated myoblasts (Day 0) and differentiated myocytes (Day 4) of C2C12 cells were treated with or without purified APN for 48 hrs. Secreted exosomes were purified and proved by antibodies against typical exosome cargos indicated. mean ± s.e.m., n = 3. *P < 0.05. (**C**–**E**) T-cad dependent APN-accumulation and exosome secretion. (**C**) Cell lysates of C2C12 cells transfected with siRNA for T-cad or negative control. mean ± s.e.m., n = 3. ***P < 0.001. (**D**) Cell lysates of C2C12 cells transfected with siRNA for AdipoR1/AdipoR2 or negative control. mean ± s.e.m., n = 3. n.s; not significant. mRNA knockdown levels were shown. mean ± s.e.m., n = 3. (**E**) siRNA for T-cad or negative control were transfected into C2C12 cells. Differentiating cells (Day 2) were treated with or without purified adiponectin for 48 hrs. Secreted exosomes were purified and proved by antibodies against typical exosome cargos indicated. mean ± s.e.m., n = 3. *P < 0.05.

**Figure 6:**
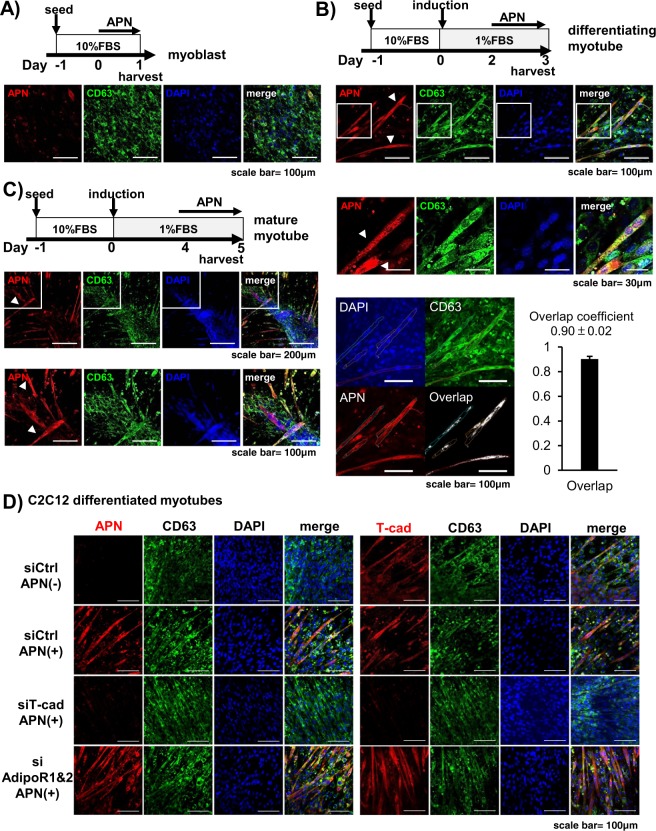
Intracellular distribution of APN. Confocal immunofluorescence micrographs of C2C12 cells. (**A**–**C**) Cells at indicated induction periods were stained with anti-adiponectin (APN; red), anti-CD63 (MVB; green). Cell nuclei were counterstained with DAPI (Nuclei; blue). Scale bars, 100 μm. (A) Undifferentiated C2C12 myoblasts. Scale bars, 100 μm. (**B**) Differentiating C2C12 myocytes. Scale bars, 100 μm; 30 μm (higher magnifications). Overlap coefficient of APN and CD63 in differentiating myocytes was calculated as described in *Methods*. Data are mean ± s.d., n = 5. (**C**) Fully differentiated C2C12 myotubes. arrow heads; differentiating myocytes. Scale bars, 200 μm; 100 μm (higher magnifications). (**D**) Differentiating C2C12 cells treated with siRNAs. siRNA for T-cad, AdipoR1/AdipoR2 or negative control were transfected into C2C12 cells. Differentiating cells (Day 2) were treated with or without purified adiponectin for 24hrs. Cell nuclei were counterstained with DAPI (Nuclei; blue). Anti-CD63 (MVB; green). Anti-adiponectin (red) for left panels and Anti-T-cadherin (red) for right panels. Scale bars, 100 μm.

To investigate APN accumulation more precisely, we examined the expression of T-cad protein during the differentiation process of C2C12 myocytes (Fig. 5A). T-cad was little expressed in undifferentiated myoblasts and gradually increased along with their differentiation into myotubes (Fig. 5A). Purified high-molecular multimeric APN, included in differentiation medium, accumulated in each stage of differentiation in accordance with the expression levels of T-cad (Fig. 5A). In agreement with such differentiation-dependent accumulation, myotubes differentiation accompanied enhanced exosome production by APN (Fig. 5B). Next, knockdown of T-cad significantly decreased APN accumulation in differentiating myotubes (Fig. 5C), while knockdown of AdipoR1 and AdipoR2 did not affect it (Fig. 5D). Furthermore, knockdown of T-cad attenuated APN-mediated increase of exosome secretion (Fig. 5E). In accordance with above quantitative studies, little accumulation of APN in undifferentiated myoblasts was seen with immunofluorescence (Fig. 6A). After starting differentiation, however, APN was selectively found in tubular myocytes undergoing differentiation (Fig. 6B). In such differentiating myocytes, APN was mainly recognized as dotted structures, highly colocalizing with exosome/multivesicular body marker CD63 (Fig. 6B, bottom panels). After 5 days of differentiation, differentiated myocytes assembled and fused into multinuclear bundles, large myotubes. Again, APN accumulated inside of differentiating myocytes (arrow heads in Fig. 6C), even in such late period of differentiation. Knockdown of T-cad but not of AdipoR1/AdipoR2 strongly attenuated such intracellular distribution of APN in differentiating myocytes (Fig. 6D).

## Discussion

Our study addressed the impact of high levels of adiponectin (APN) on injury-induced muscle regeneration and the role of T-cad mediating the function of APN. APN overexpression in mice decreased necrotic region and increased regenerating myotubes in angiotensin II (AII)-infusion mice. Such enhanced regeneration by excess APN was also observed in APN null mice, but not in T-cad null mice. T-cad was decreased by muscle injury and gradually restored with muscle regeneration. The ectopic accumulation of APN in muscle well correlated with this. APN accumulated on plasma membrane of myofibers both in mice and human. Importantly, APN accumulated in endosome-like structures positive for CD63 inside of regenerating myotubes but not in unaffected intact myofibers nor in necrotic ones. Such intracellular localization specifically in regenerating myofibers coincided with the changes of T-cad localization during regeneration. Purified high-molecular multimeric APN similarly accumulated intracellularly and colocalized with a multivesicular bodies/exosomes marker CD63 in differentiating myocytes but not undifferentiated myocytes. In agreement with these differentiation-dependent endosomal accumulation, APN-mediated exosome production was also differentiation dependent and attenuated by T-cad knockdown experiment.

It was reported that elevated circulating APN levels were an independent markers of both myocardial infarction (MI) and all-cause mortality in male patients undergoing coronary angiography^37^. Such inverse correlation with circulating APN levels has been also reported on muscle weakness in elderly people^31,32^, muscle mass and strength in elderly heart failure patients^33^. Here we have revealed that high circulating APN levels in mice were not inhibitory but promotive for muscle regeneration at least under high angiotensin II levels, suggesting that above mentioned higher circulating APN in elderly patients especially with heart failure may not be causative for their associated muscle weakness and loss of muscle mass. Our conclusion agrees with the notions obtained in Duchenne muscular dystrophy model mice, where overexpression of APN decreased muscle damage^38^ and loss of APN caused lower muscle force/endurance^39^.

It was reported that APN levels are positively associated with age, even after adjustment for visceral adiposity^40,41^. Regulation of circulating level of APN has not been fully understood. A decrease in its clearance in the kidney may be the cause of high levels of APN in the elderly^42,43^. Circulating APN decreases in visceral adiposity and insulin-resistance, likely because of decreased production and secretion from white adipose tissues. Conversely, leanness is associated with higher circulating APN. The production from increased bone marrow adipose tissue in addition to the increased production of APN from white adipose tissue contributes to hyperadiponectinemia in leanness^44^. Bone marrow adipose tissue increases with aging and may account for higher level of APN in the elderly. We and others recently reported that loss of T-cad in mice resulted in more than three-fold increase of plasma APN^19,21^. SNP around T-cad gene was reported to associate with plasma APN levels in human beings^22–27^. Thus, if the T-cad expression is attenuated by aging, it may contribute to higher APN levels in elderly. In our study, loss of APN did not significantly affect muscle regeneration, suggesting physiological level of APN has little role on such short-term regeneration reaction. We employed adenovirus-mediated overexpression of APN to delineate whether experimentally high APN (>10-fold increase) is deleterious or promotive for muscle regeneration in mice. The results suggested that high APN is promotive for muscle regeneration through T-cad.

Here we employed AII-infusion to mimic aging and chronic heart failure condition^11,12,36^. This treatment attenuated regeneration, otherwise it would be difficult to see the improvement of muscle regeneration in C57BL/6J mice, which have a better regeneration potential^45^. In agreement with those notions, AII-infusion, also in this study, up-regulated Axin2 expression, a downstream gene of the aging-promoting Wnt/β-catenin signaling pathway^36^. APN overexpression did not attenuate Axin2 expression, suggesting it had no significant effect on this signaling. However, AII-signaling on capillary endothelium in muscle tissue could be significantly attenuated by APN as we reported on cardiac tissue in AII induced cardiac hypertrophy model^46,47^. Along with this context, our current study has not evaluated the importance of angiogenesis after muscle injury. It was reported that APN promoted angiogenesis in a model of hind limb ischemia^20^. Because angiogenesis is also important for tissue repairing, our study cannot exclude that angiogenesis enhancement by APN resulted in faster muscle regeneration.

T-cad is abundantly expressed in skeletal muscle in addition to vascular endothelium and heart. We identified regeneration-induced unique intracellular localization of APN specifically in regenerating myofibers and colocalization with CD63, an exosome and multivesicular body (MVB) marker. We recently reported that APN accumulated in multivesicular bodies and stimulated exosome biogenesis, which accompanied excretion of unnecessary or harmful materials such as ceramide^30^. APN rescued the impaired regeneration caused by AII infusion. This required the presence of T-cad. Muscle regeneration accompanied the change of distribution of APN from cell surface into CD63-positive endosomes, which possibly reflects its accumulation into MVBs. MVBs are the endosomes producing exosomes. We recently reported that APN accumulated in MVBs in cultured endothelial cells and *in vivo* aortic endothelial cells^30^. Collectively, our study clearly indicated that T-cad mediated the promoting function of APN in muscle regeneration, and T-cad in regenerating myofibers had some roles in this process.

## Methods

### Study approval

Human iliopsoas muscle autopsy specimens were obtained after written informed consent for postmortem investigation was obtained from the subject prior to his or her death or afterward from the subject’s relatives. The experimental protocol was approved by the Ethics Review Committee for Animal Experimentation of Osaka University School of Medicine and also conforms to the Guide for the Care and Use of Laboratory Animals published by the United States National Institute of Health.

### Animal procedure

C57BL6/J male mouse was purchased from CLEA Japan. Adiponectin (APN) KO and T-cadherin (T-cad) KO mice had already been generated. All APN KO and T-cad KO mice were bred on a C57BL/6J background. Mice were maintained at 22 °C in a 12:12 h light-dark cycle (lights of from 8:00 AM to 8:00 PM).

Angiotensin II (AII) was infused as described previously^47^. Briefly, AII (Sigma) was dissolved in 0.01 M acetic acid. Mice were anesthetized and implanted osmotic minipumps (Alzet^TM^ mini-osmotic pump model 2002, Durect Corp.) containing 2.4 mg/kg/day of AII (Sigma) dissolved in 0.01 M acetic acid, in midscapular region of mice at 12–16 weeks of age. To induce muscle regeneration, 50 µL of 10 µM cardiotoxin (CTX) (Latoxan) was injected to each tibialis anterior (TA) muscle. Supplementation of APN was performed as described previously^47^. Adenovirus expressing the full-length mouse APN (Ad-APN) or the β-galactosidase (Ad-βgal) was prepared using the Adenovirus Standard Purification Virakit^TM^ (Virapur). Then, they were injected to mice via the tail vein at 4 days before cardiotoxin injection and pump implantation. At sacrifice, mice were anesthetized by intraperitoneal injection of medetomidine (0.3 mg/kg body weight), midazolam (4 mg/kg body weight), and butorphanol (5 mg/kg body weight) and transcardially perfused with cold saline to wash out circulating APN. Serum APN levels were measured by ELISA (Otsuka Pharmaceutical Co.).

### Exosome preparation

Exosome isolation from the cell culture supernatant was performed as described previously^30^, with some modifications. Briefly, differentiated C2C12 cells were cultured with DMEM containing indicated concentration of exosome-free FBS for 48 hours. Then, the conditioned medium was collected and centrifuged at 800 × g for 10 minutes to deplete floating cells, and at 10,000 × g for 30 minutes to remove cell debris. For exosome isolation, the supernatant was ultracentrifuged at average 110,000 × g for 2 hours, followed by a washing step of the exosome pellet with Dulbecco’s phosphate-buffered saline with calcium and magnesium (PBS(+)) at average 110,000 × g for 2 hours (TLA100.1 rotor, Beckman Coulter). The exosome pellets were directly solubilized in Laemmli sample buffer. For comparative analysis, exosomes were collected from equivalent amounts of culture medium, conditioned by equivalent numbers of cells.

### Western blotting

Exosomes or whole cell lysates were loaded onto 4–20% gradient SDS-PAGE gels (Bio-Rad) and transferred onto nitrocellulose membranes. The membranes were blocked with Block-One^TM^ blocking reagent (Nacalai Tesque) and then incubated with primary antibodies using Can Get Signal^TM^ solution 1 (TOYOBO) overnight at 4 °C and followed by incubation with secondary antibodies conjugated with HRP using Can Get Signal^TM^ solution 2 (TOYOBO) for 60 minutes at room temperature. The following primary antibodies were used: goat polyclonal anti-adiponectin (AF1119, R&D); goat polyclonal anti-T-cadherin (AF3264, R&D); rabbit monoclonal anti-α-tubulin (11H10, Cell Signaling); rat monoclonal anti-mouse CD63 (clone R5G2, MBL); rabbit polyclonal anti-syntenin (ab19903, abcam); rabbit polyclonal anti-Tsg101 (ab125611, abcam); goat polyclonal anti-MFG-E8 (AF2805, R&D); mouse monoclonal anti-Alix (ab117600, abcam); rabbit polyclonal anti-Nox2/gp91 phox (ab80508, abcam). CD63 was detected under non-reducing conditions. Chemiluminescence signals developed with Chemi-Lumi One Super^TM^ (Nacalai Tesque) were visualized by ChemiDoc Touch^TM^ and quantitated using Image Lab software (Bio-Rad).

### Muscle fixation and histological analysis

Formalin-fixed, paraffin-embedded human autopsy specimens were deparaffinized, sectioned (2-μm thick) using a cryostat (Leica Microsystems) and stained with H&E. Mouse tibialis anterior (TA) muscles were resected and frozen in isopentane cooled in liquid nitrogen. Frozen tissue was sectioned (10-μm thick) and stained with H&E. Myofiber area and necrotic area were quantified with a BZ-X700 microscope and its software (Keyence), according to the following procedure. For myofiber area quantification, we isolated eosin red stained regenerating myofibers from whole sectional image, using color extraction function of BZ-X analyzer. Then, the ratio of the obtained myofiber area to total sectional area was calculated. For necrotic area analyses, we first defined necrotic area as containing more than three clustering of necrotic fibers. Necrotic fibers can be identified as round-shaped, eosin stained myofibers with peripherally located nuclei. They can accompany crescent-shaped, centrally nucleated, regenerating myocytes emerging around them. They are distinguished from intact fibers by surrounding infiltrating immunocytes or proliferating interstitial tissues. Also using BZ-X analyzer, above defined necrotic areas are extracted from whole sectional image. Then, the ratio of the obtained necrotic area to total sectional area was calculated.

### Immunofluorescence

Human muscle sections (2-μm thick) were blocked with 3% BSA and incubated with the following primary antibodies at 4 °C overnight: mouse-anti human APN (1:100, abcam), goat-anti human T-cad (1:100; R&D), followed by secondary staining. Mouse muscle cryosections (6-μm thick) were fixed with acetone for 10 minutes at −20 °C. Specimens were blocked with Protein-block^TM^ serum free reagent (Dako), and then incubated with the following primary antibodies at 4 °C overnight: goat-anti APN (1:100; R&D), mouse-anti mouse eMyHC (1:2; DSHB), rat-anti laminin α2 (1:400; Alexis, San Diego, CA, USA), rat-anti mouse CD63 (1:100; MBL), goat-anti human T-cad (1:100; R&D), followed by secondary staining. The M.O.M. kit^TM^ (Vector Laboratories) was used when mouse primary antibody was used for staining. Specimens were counterstained with 4′,6-Diamidino-2-Phenylindole (DAPI) (Invitrogen). Microscopy analysis was performed using an Olympus FV1200D confocal laser scanning microscope system (Olympus) for APN and T-cad localization analysis. For quantification of eMyHC positive area, signals were detected and analyzed using a BZ-X700 microscope and its software (Keyence). Colocalization analysis was carried out by calculating overlap coefficients of two signals using FLUOVIEW software (Olympus).

### Cell culture and cellular immunofluorescence staining

C2C12 mouse myoblasts were cultured in DMEM with 10% FBS, 100 U/mL penicillin, and 100 µg/mL streptomycin. Myogenic differentiation was induced by culture in collagen-I coated plates with supplemented with 1% FBS. For knockdown experiments, siRNAs used are as follows; siRNA for mouse T-cadherin (s63759, Ambion); mouse AdipoR1 (s91210, Ambion); mouse AdipoR2 (s86687, Ambion). AdipoR1 and AdipoR2 were cotransfected. For western blot analysis, cells were harvested 48 hours after seed, and 2^nd^ and 4^th^ day of differentiation. For immunofluorescence staining, APN-containing medium commenced on 24hrs after the indicated timing, then cells were harvested. Cells seed on coverslips were fixed with periodate-lysine-paraformaldehyde (PLP) for 30 minutes and incubated with 3% w/v BSA and 0.3% w/v Triton X-100 in Dulbecco’s phosphate-buffered saline free of calcium and magnesium (PBS) for 60 minutes. Cells were then incubated with the following primary antibodies overnight at 4 °C; goat-mouse anti APN (1:100; R&D), rat-anti mouse CD63 (1:100; MBL), followed by incubation with Alexa Flour-conjugated secondary antibodies for 60 minutes at room temperature. Microscopy analysis was performed using an Olympus FV1200D confocal laser scanning microscope system (Olympus).

### Purification of Adiponectin

APN purification was performed as previously described^29^. Briefly, serum from APN KO mice treated with Ad-APN was applied onto T-cad-Fc conjugated with Protein G sepharose (GE healthcare). APN was eluted with 5 mM EDTA.

### Quantitative RT-PCR

Total RNA was isolated from mouse tissues as described previously using the RNA STAT-60 (Tel-Test) according to the protocol supplied by the manufacturer. Purified RNA was processed with DNase I (Invitrogen). First-strand cDNA was synthesized using the EvoScript Universal cDNA Master (Roche Applied Science). Real-time quantitative PCR amplification was conducted with the Light Cycler 480 (Roche Applied Science) using Light Cycler 480 SYBR Green I Master (Roche Applied Science) according to the protocol recommended by the manufacturer. The sequences of primers used for real-time PCR were as follows:

*hprt*, *FW* 5′-CTTTGCTGACCTGCTGGATTACAT-3′

*Rv* 5′-GTCCCCCGTTGACTGATCATTAC-3′

*emyhc*, *FW 5*′*-AAAAGGCCATCACTGACGC-*3′

*Rv 5*′*-CAGCTCTCTGATCCGTGTCTC-3*′

*col1a1*, *FW 5*′*-AGACATGTTCAGCTTTGTGGAC-3*′

*Rv 5*′*-GCAGCTGACTTCAGGGATG-3*′

*axin2*, *FW 5*′ *-CCATGACGGACAGTAGCGTA-3*′

*Rv 5*′*-GCCATTGGCCTTCACACT-3*′

*gapdh*, *FW 5*′*-GCACAGTCAAGGCCGAGAAT-3*′

*Rv 5*′*-GCCTTCTCCATGGTGGTGAA-3*′

*36B4*, *Fw 5*′*-GGCCAATAAGGTGCCAGCT-3*′

*Rv 5*′*-TGATCAGCCCGAAGGAGAAG-3*′

*AdipoR1*, *FW 5*′*- AATGGGGCTCCTTCTGGTAAC-3*′

*Rv 5*′*- GCAGACCTTATACACGAACTCC-3*′

*AdipoR2*, *FW 5*′*-GGAGTGTTCGTGGGCTTAGG-3*′

*Rv 5*′*- GCAGCTCCGGTGATATAGAGG-3*′.

### Statistics

Data are expressed as mean ± s.e.m. *P* values were calculated by Student’s t-test, except for Fig. 2A by Dunnett’s test and Figs 3B, 5A, and S2A,B by Tukey’s test. Overlap coefficients were expressed as mean ± s.d. in Figs 2E,6B.

## Supplementary information

Figure S1, S2, S3, S4

## Data Availability

The datasets generated during and/or analyzed during the current study are available from the corresponding author on reasonable request.
