# Author Correction: Adiponectin promotes muscle regeneration through binding to T-cadherin

**DOI:** 10.1038/s41598-020-66545-1

**Published:** 2020-07-17

**Authors:** Yoshimitsu Tanaka, Shunbun Kita, Hitoshi Nishizawa, Shiro Fukuda, Yuya Fujishima, Yoshinari Obata, Hirofumi Nagao, Shigeki Masuda, Yuto Nakamura, Yuri Shimizu, Ryohei Mineo, Tomoaki Natsukawa, Tohru Funahashi, Barbara Ranscht, So-ichiro Fukada, Norikazu Maeda, Iichiro Shimomura

**Affiliations:** 1 Department of Metabolic Medicine, Graduate School of Medicine, Osaka University, Osaka, Japan; 2 Department of Adipose Management, Graduate School of Medicine, Osaka University, Osaka, Japan; 3 Department of emergency & intensive care, Yodogawa Christian Hospital, Osaka, Japan; 4 Department of Metabolism and Atherosclerosis, Graduate School of Medicine, Osaka University, Osaka, Japan; 5 Sanford Burnham Prebys Medical Discovery Institute, NIH-designated Cancer Center, Development, Aging and Regeneration Program, La Jolla, CA USA; 6 Laboratory of Molecular and Cellular Physiology, Graduate School of Pharmaceutical Sciences, Osaka University, Osaka, Japan

Correction to: *Scientific Reports* 10.1038/s41598-018-37115-3, published online 09 January 2019

The Supplementary Information file that accompanies this Article contains an error in Figure S3 C) APN KO All (+) where an image is omitted and other images are incorrectly duplicated from Figure S3 B) WT All (+). The correct Figure S3C appears below as Figure 1.

**Figure 1:**
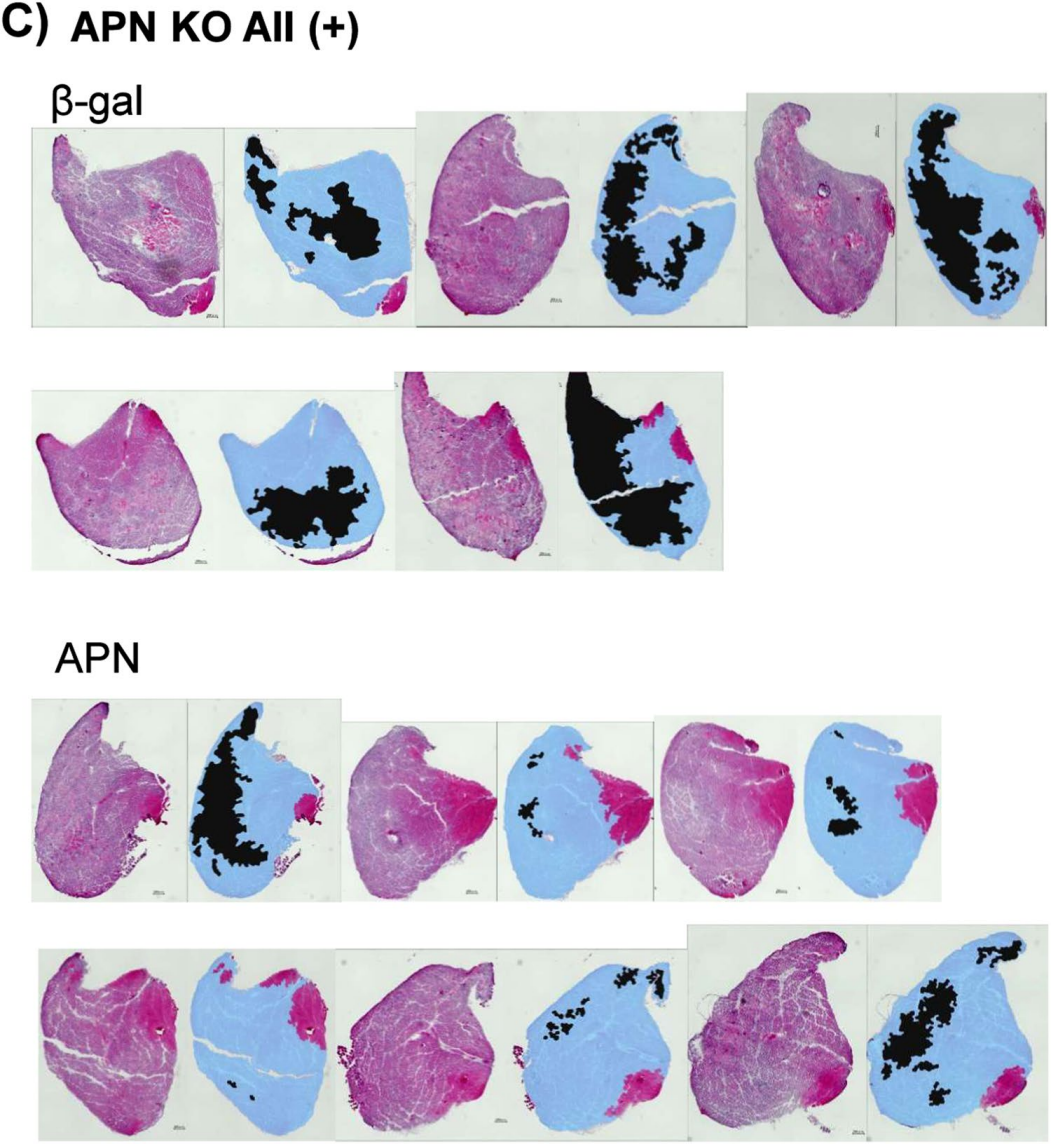
.

