# Supplementary material for: Adiponectin promotes muscle regeneration through binding to T-cadherin
Source: Sci Rep. 2019 Jan 9;9:16. doi: 10.1038/s41598-018-37115-3 (PMC6327035; doi:10.1038/s41598-018-37115-3)

Supplemental Data

Adiponectin promotes muscle regeneration through binding to T-cadherin.

Yoshimitsu Tanaka,[1] Shunbun Kita,[*,1,2] Hitoshi Nishizawa,[1] Shiro Fukuda,[1] Yuya Fujishima,[1] Yoshinari Obata,[1] Hirofumi Nagao,[1] Shigeki Masuda,[1] Yuto Nakamura,[1] Yuri Shimizu,[1] Ryohei Mineo,[1] Tomoaki Natsukawa,[3] Tohru Funahashi,[1,4] Barbara Ranscht,[5] So-ichiro Fukada,[6] Norikazu Maeda,[1,4] and Iichiro Shimomura[1]

Contents

Fig. S1. Higher magnifications of Fig. 2C.

Fig. S2 APN overexpression and angiotensin II-signaling.

Fig. S3 Raw images and identified necrotic regions of Fig. 3 and Fig. 4

Fig. S4 Raw images of western blots.

Supplemental Figure legends

Fig. S1. Higher magnifications of Fig. 2C.

Fig. S2. APN overexpression and angiotensin II-signaling. (A) Serum concentration of APN at 7 days after CTX-injury in Figure 3A. mean±s.e.m., n=3 (AII, β-gal, APN), n=6 (AII+, β-gal APN), ***P*<0.01, *** *P* <0.001, Turkey's test. (B) Axin2 expression levels 7 days after CTX-injury. mean±s.e.m., n=2 (AII-, APN), n=3 (AII-, β-gal), n=6(others), n.s; not significant; * *P* <0.05, Turkey's test. (C-F) Canonical angiotensin II signaling at 7 days after CTX-injury; ERK phosphorylation (C); p38MAP-kinase phosphorylation (D); NFκB p65 subunit phosphorylation (E); NOX2 protein (F). mean±s.e.m., n=6. Not statically significant (C-F).

Fig. S3. Raw images and identified necrotic regions of Fig. 3 and Fig. 4. Identified necrotic region area were colored black, regenerating areas were colored blue. Sum of necrotic region area and regenerating area was defined as total area (total affected area by CTX injection).

Fig. S4 Raw images of western blots. Full images of western blot including every blots calculated in bar-graphs were shown.

Tanaka et al. Fig. S1

Higher magnifications of Fig. 2C

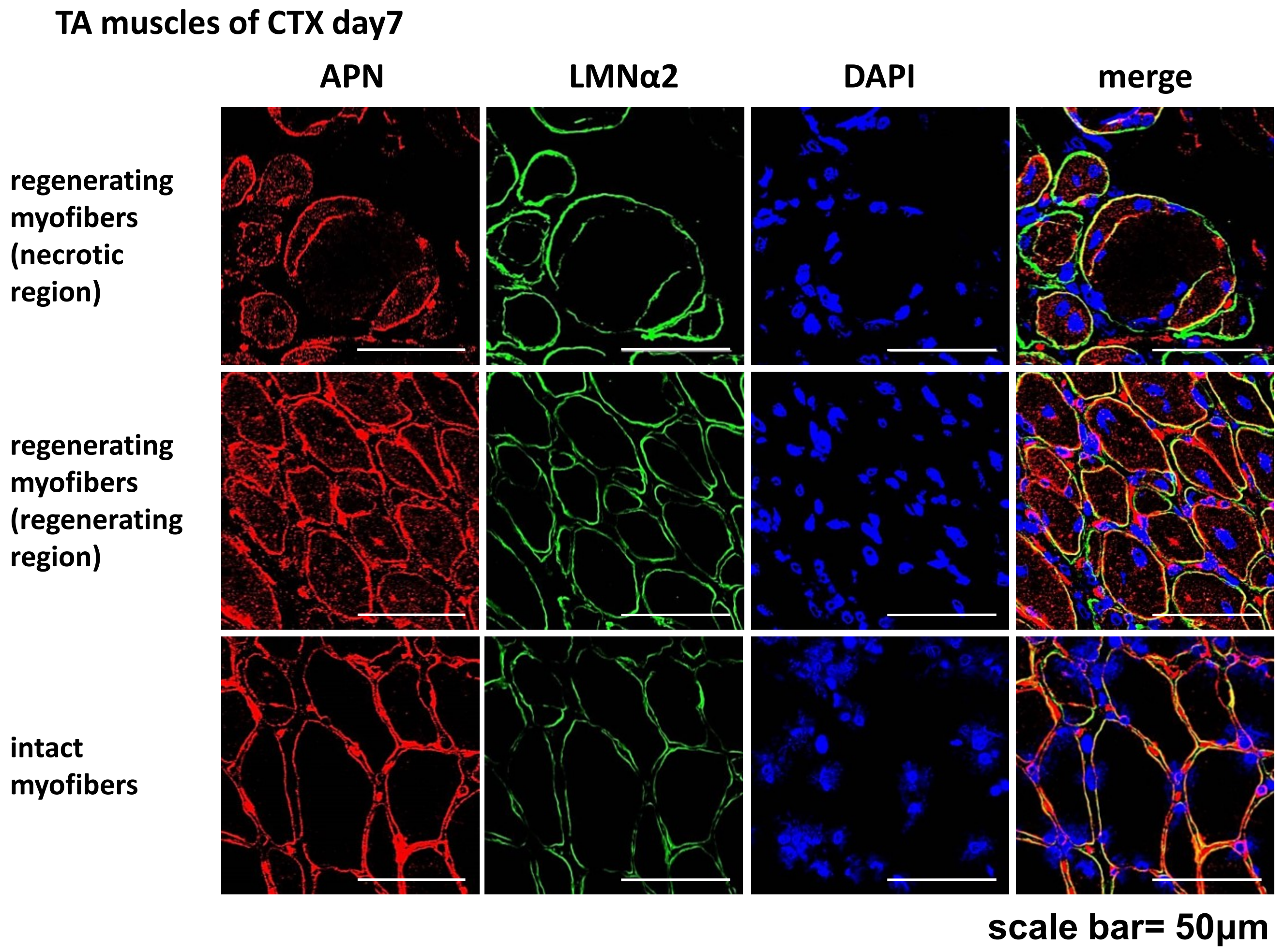

Tanaka et al. Fig. S2

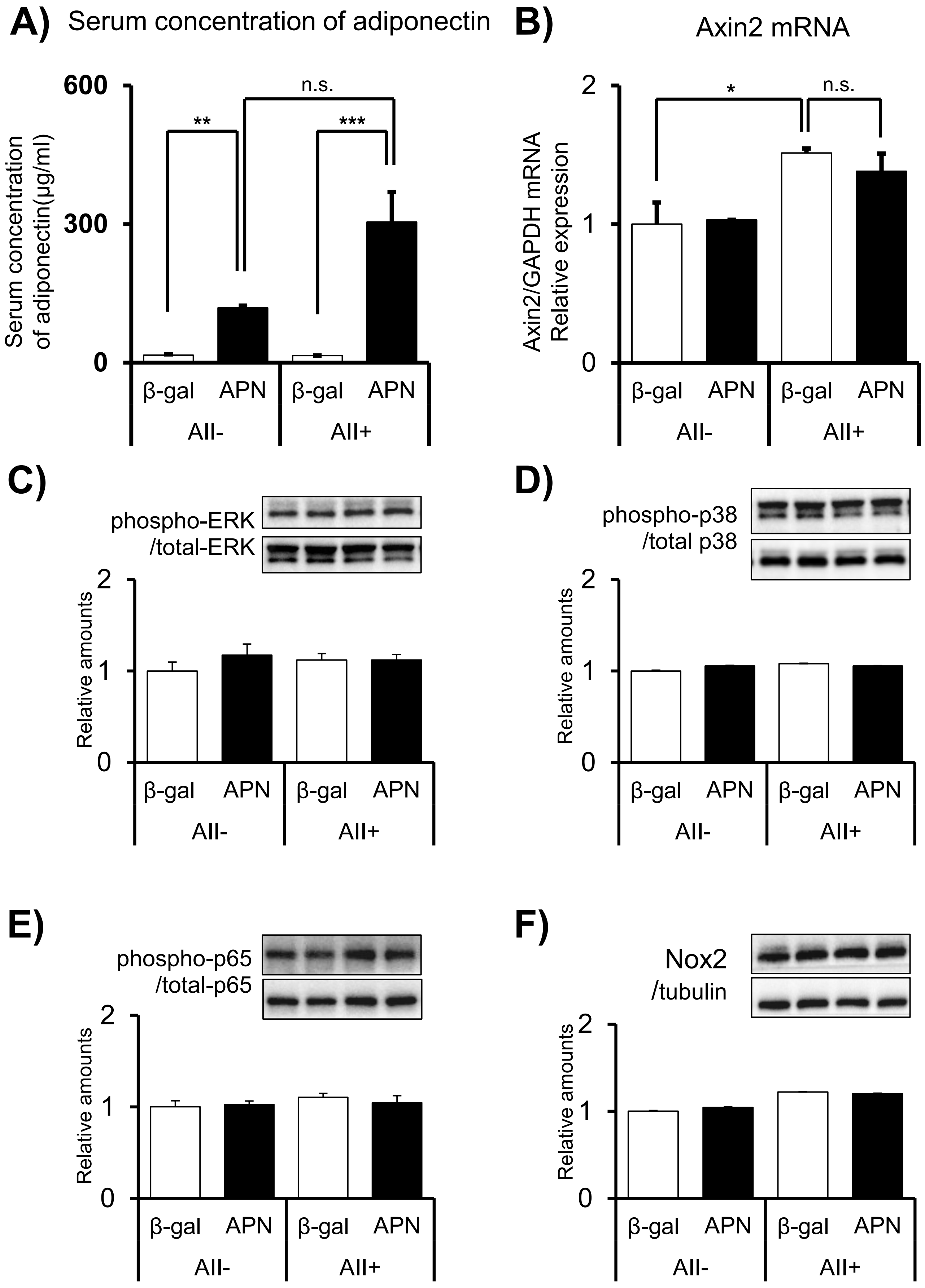

Tanaka et al. Fig. S3

A) **WT AII (-)**

β-gal

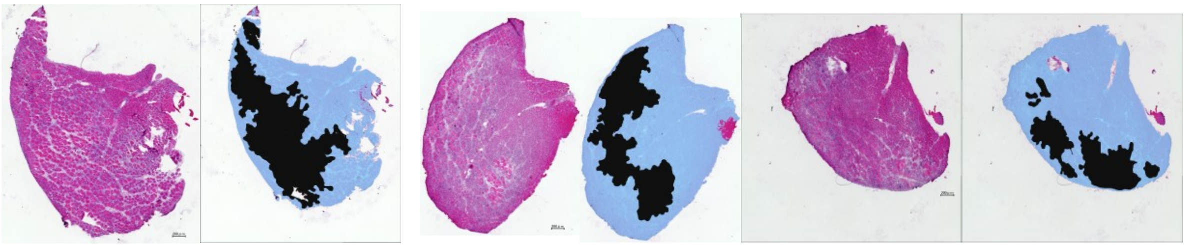

APN

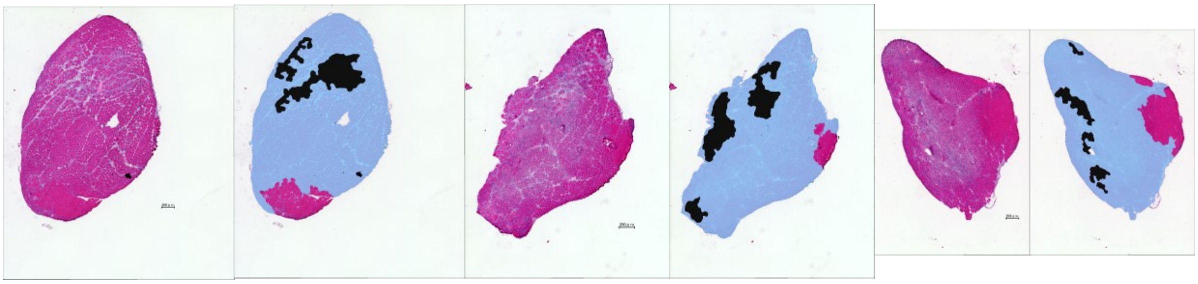

B) WT AII (+)

β-gal

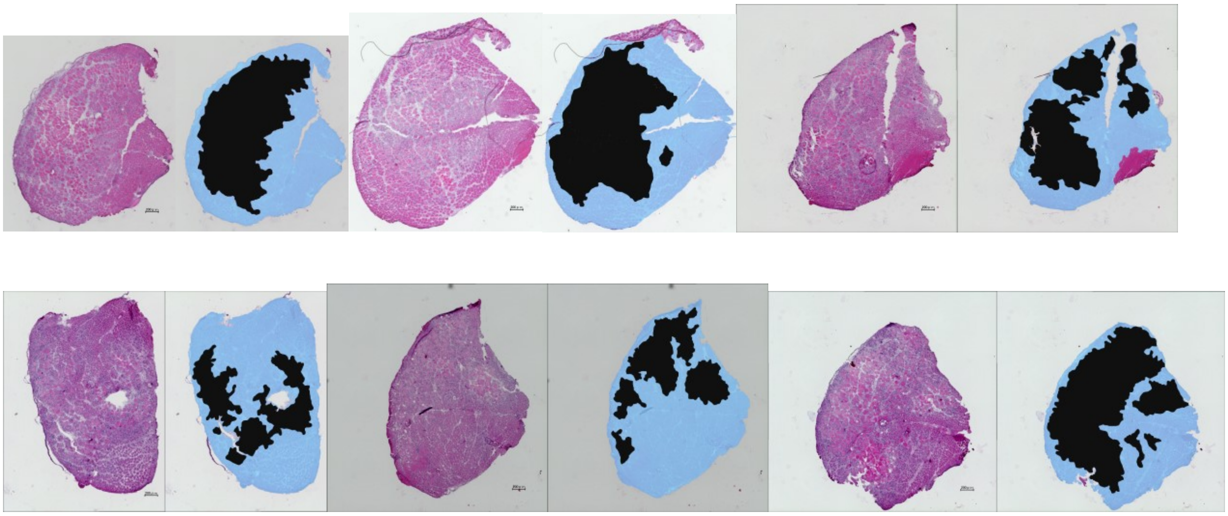

APN

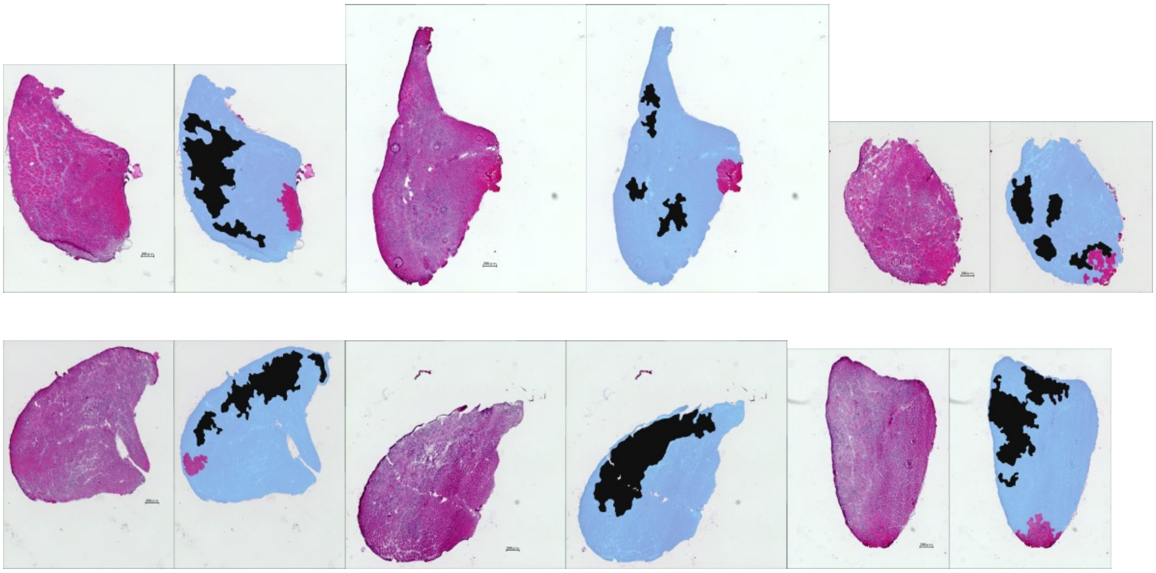

C) APN KO AII (+)

β-gal

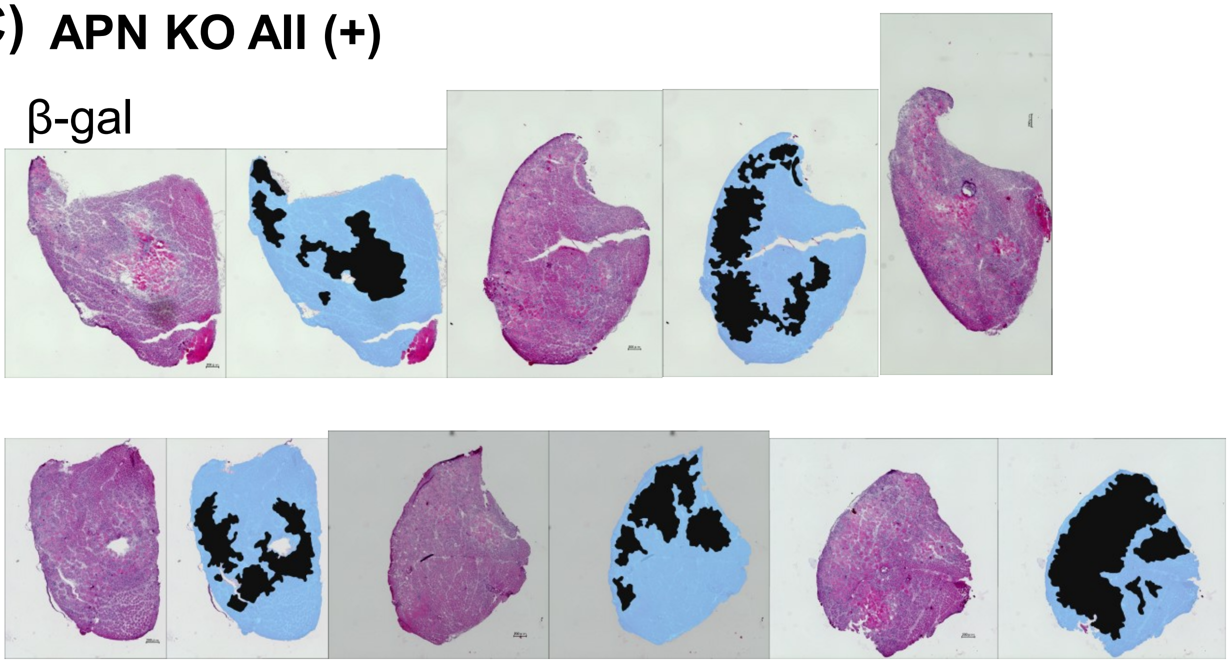

APN

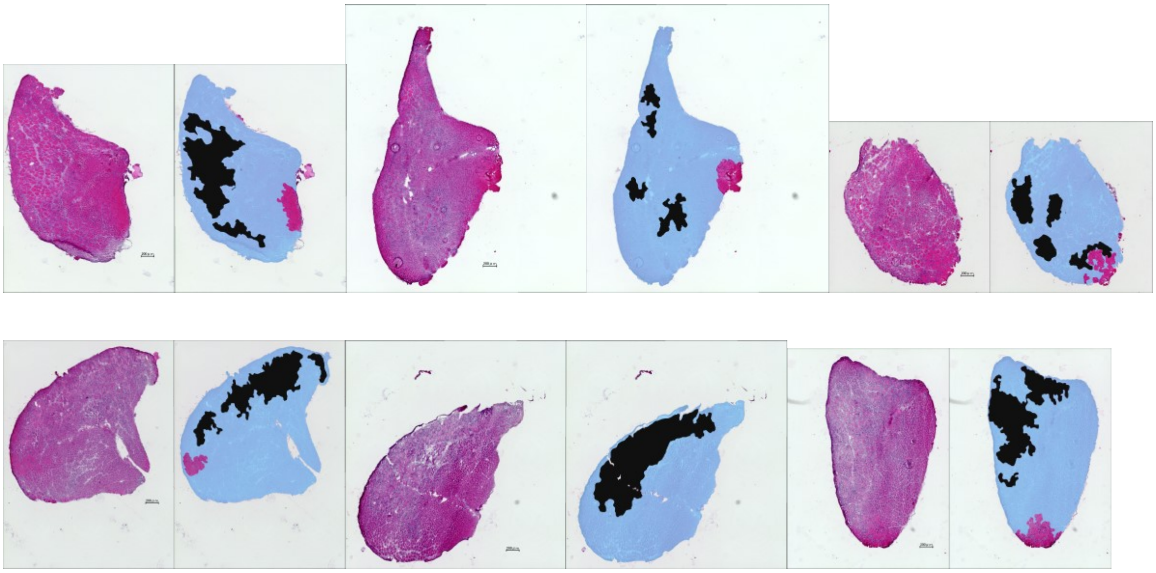

D) T-cad KO AII (+)

β-gal

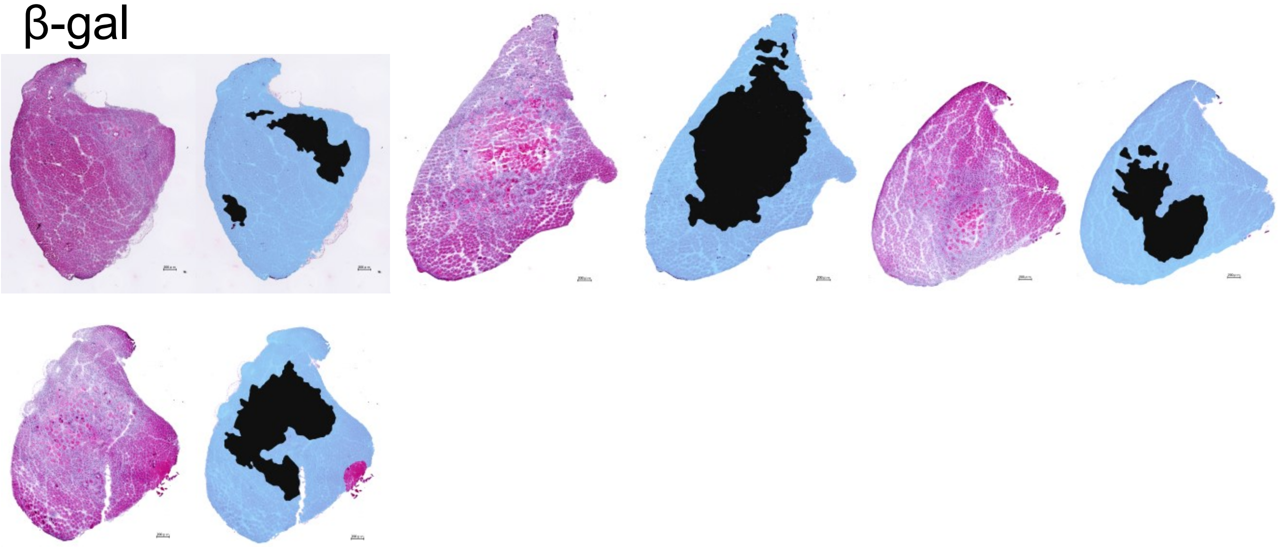

APN

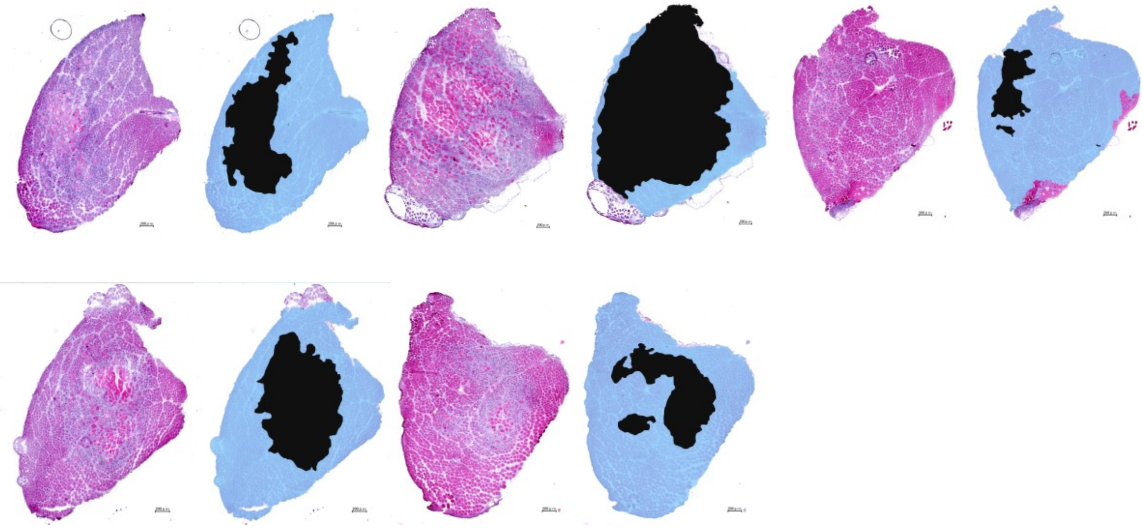

Tanaka et al. Fig. S4

Raw blots of Fig. 2A

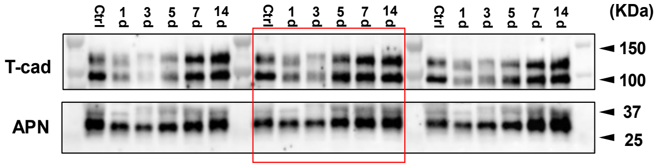

Raw blots of Fig. 5B

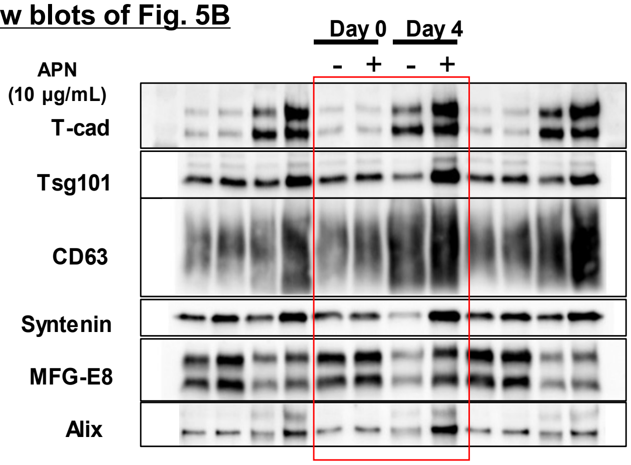

Raw blots of Fig. 5A

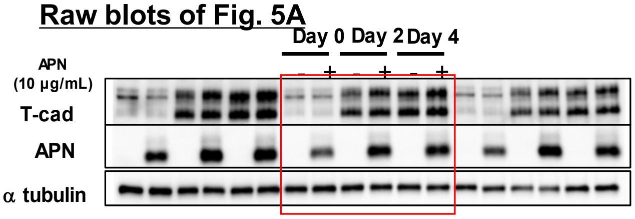

Raw blots of Fig. 5C

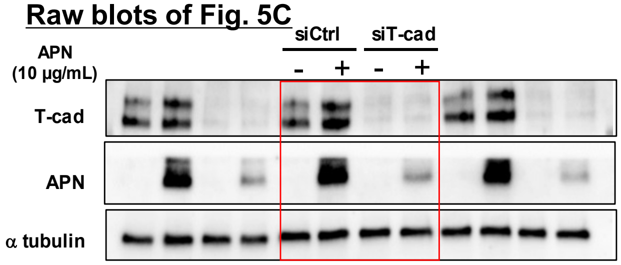

Raw blots of Fig. 5D

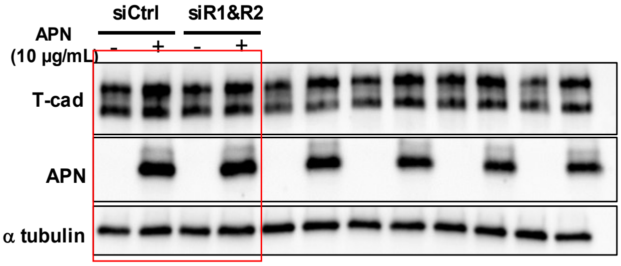

Raw blots of Fig. 5E

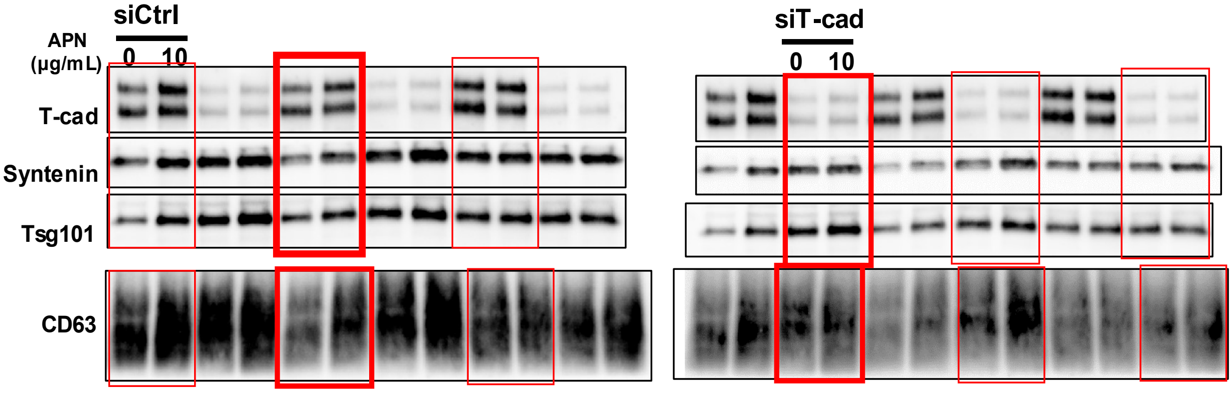

Supplement: Supplementary file 1 — Figure S1, S2, S3, S4 [file 41598_2018_37115_MOESM1_ESM.pdf]
